# A Fatal Case of Basidiobolomycosis

**DOI:** 10.7759/cureus.78678

**Published:** 2025-02-07

**Authors:** Abdulellah I Aleissa, Abdulaziz Kadasa, Maram Alzain, Khadijah Abdulhaleem, Luluwah Almubarak

**Affiliations:** 1 Dermatology, King Abdulaziz University Faculty of Medicine, Jeddah, SAU; 2 College of Medicine, King Abdulaziz University, Jeddah, SAU; 3 Dermatology, Prince Sultan Military Medical City, Riyadh, SAU; 4 Central Military Laboratory and Blood Bank, Prince Sultan Military Medical City, Riyadh, SAU

**Keywords:** basidiobolomycosis, fungal, fungal diseases, fungal infections, skin infections, skin lesions

## Abstract

Basidiobolomycosis is a rare fungal infection caused by *Basidiobolus ranarum*. *B. ranarum* is present worldwide but is more common in tropical and subtropical regions. Typically, it infects exposed surfaces of the body causing subcutaneous nodules, sinus, and nasal infection; however, it rarely causes a soft tissue mass. Histopathological examination is mandatory to make a diagnosis of basidiobolomycosis. Here, we report a case of a five-year-old boy who presented with skin subcutaneous nodules and retroperitoneal and retroaortic soft tissue masses. The histopathological examination confirmed the diagnosis of basidiobolomycosis.

## Introduction

Basidiobolomycosis is a rare disease caused by the fungus *Basidiobolus ranarum* [1]. The first human case of subcutaneous mycosis was recognized in Indonesia in 1956 [2]. The subcutaneous form of the disease has been reported mainly from the tropical areas of Africa, Asia, Latin America, and Arizona [3]. Although it typically infects exposed surfaces of the body causing subcutaneous nodules, sinus, and nasal infection, it rarely causes a soft tissue mass [4]. Tissue biopsy is mandatory for the diagnosis of basidiobolomycosis [5].

## Case presentation

**Figure 1:**
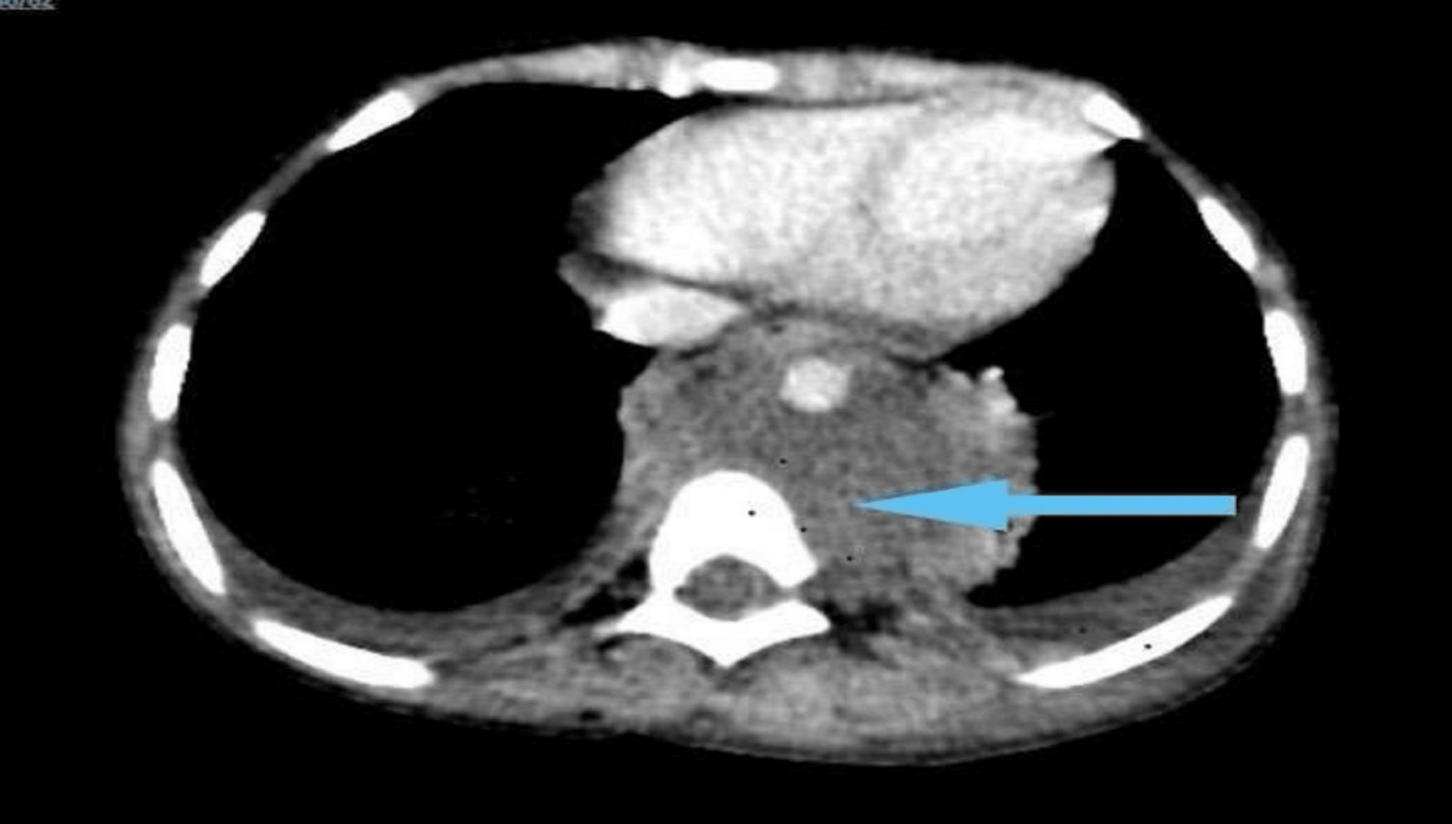
CT scan showing retroperitoneal and retroaortic soft tissue mass.

A five-year-old male presented to the emergency department with a complaint of high-grade fever associated with weight loss, shortness of breath, and severe lower back pain for two months. Two weeks before admission the patient’s mother had noticed two skin nodules over his abdomen and thigh. The patient underwent a CT scan that showed retroperitoneal and retroaortic soft tissue masses (Figure 1).

**Figure 2:**
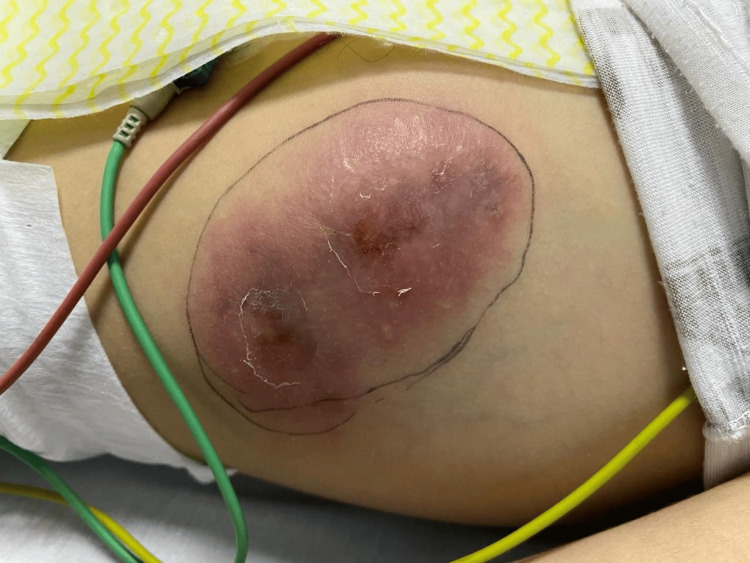
Well-demarcated erythematous, tender, hot, and firm indurated plaque over the left side of the chest measuring 9 × 3 cm.

**Figure 3:**
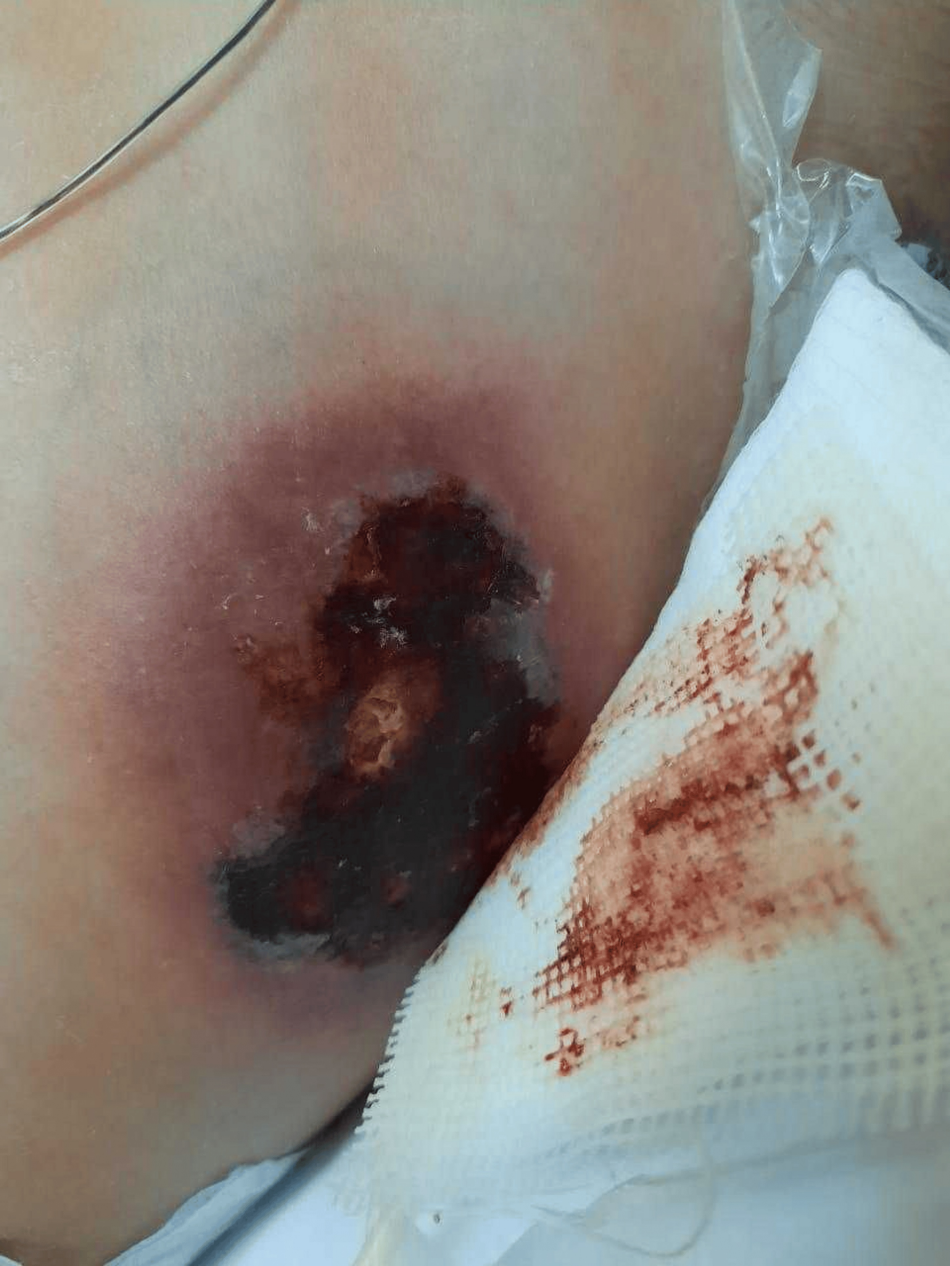
Well-demarcated erythematous, tender, hot, and firm indurated plaque over the left thigh measuring 2 × 2 cm.

On examination, the patient looked pale, inactive, and lethargic. The skin examination revealed two well-demarcated erythematous, tender, and firm indurated plaques over the left side of the lower chest measuring 9 × 3 cm (Figure 2) and over the left thigh measuring 2 × 2 cm (Figure 3).

**Figure 4:**
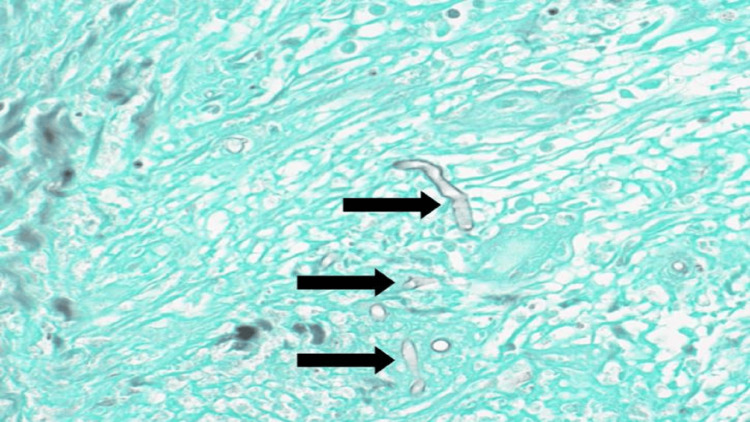
Extensive necrosis associated with mixed inflammation and infestation by numerous broad and branching fungal hyphae (Grocott-Gömöri’s methenamine silver stain, 40× magnification).

**Figure 5:**
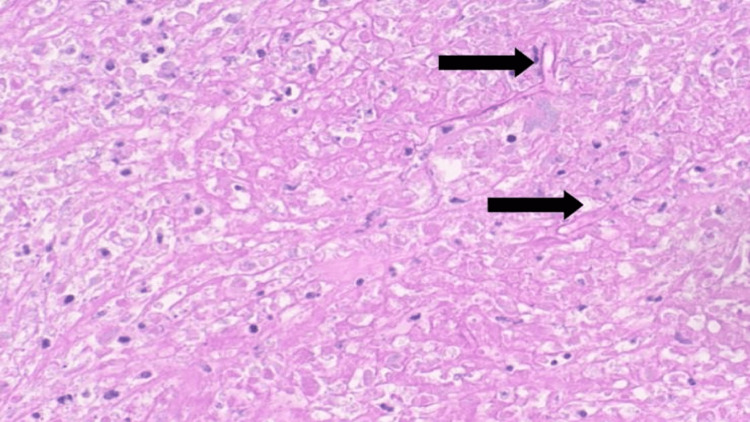
Extensive necrosis associated with mixed inflammation and infestation by numerous broad and branching fungal hyphae (periodic acid-Schiff stain, 40× magnification).

No lymph node swelling was observed. The patient was admitted to the hospital to rule out malignancy, especially neuroblastoma. Skin and soft tissue deep biopsies were performed. The histological examination of skin biopsy showed stratified squamous epithelium underlined by dermis and subcutaneous tissue, both were heavily infiltrated by mixed inflammatory infiltrate. Fungal elements were identified within the tissue with special stains for fungi. The histological examination of the retroperitoneal mass biopsy showed marked necrotizing inflammation with fungal elements. Necrotic material with mixed inflammation mainly neutrophils and scattered eosinophils was observed. Fungal microorganisms with broad thin-walled hyphae surrounded by intense eosinophilic material (Splendore-Hoeppli phenomenon) were noted. Special stains such as Grocott-Gömöri’s methenamine silver stain (Figure 4) and periodic acid-Schiff were positive for fungi (Figure 5).

No malignancy was seen in the examined material. The overall findings were suspicious for basidiobolomycosis. The patient was started on meropenem, teicoplanin, caspofungin, and liposomal amphotericin B.

Chronic granulomatous disease (CGD) was suspected. Whole exome sequencing showed a homozygous pathogenic variant in the *NCF1* gene. The genetic diagnosis of autosomal recessive CGD type 1 was confirmed.

The patient deteriorated rapidly and was admitted to the pediatric intensive care unit due to respiratory distress and pleural effusion. He was in critical condition. After a few days, the patient developed acute renal failure. He was started on hemodialysis and intubated with inotropic support. The patient became bradycardic and was resuscitated unsuccessfully and later declared dead.

## Discussion

B. ranarum causes zygomycosis characterized by skin and subcutaneous tissue infections [1]. Basidiobolomycosis was first described by Joe et al. in Indonesia [2]. Being a saprophytic fungus, *B. ranarum* is typically found in soil, decaying plants, and gastrointestinal tracts of insects, rodents, fish, and amphibians [4]. B. ranarum infection is endemic to the tropics and subtropical regions [4].

The major load of patients with cutaneous basidiobolomycosis is from the tropical regions of Africa, Asia, Latin America, and Arizona. An arid climate facilitates the growth of the fungus [3]. Even though *B. ranarum* is widely distributed, basidiobolomycosis in humans is rare. The mode of transmission is not exactly known, and it can infect humans through insect bites and trauma [4].

Compared to other fungal infections, *B. ranarum* infection occurs predominantly in immunocompetent children and adults, with males affected more often than females. The exposed parts of the body are involved in most of the infections, with only a few cases of pulmonary, gastrointestinal, and head and neck involvement [6]. Usually, cutaneous infections present as a single, painless, firm subcutaneous nodule, developing at the place of exposure to the fungus. The skin over the nodule becomes purplish in color. Lymphedema can also occur in the affected limb. Gastrointestinal basidiobolomycosis is an uncommon but serious infection of *B. ranarum* [4]. Cases of basidiobolomycosis mimicking inflammatory bowel disease [7], fistulizing Crohn's disease [8], and colon cancer [9] have been reported. B. ranarum infections generally have a good prognosis unless it is extensive and spread to the mediastinum, bowel, scrotum, and penis, in which case it can sometimes be fatal [5].

Histological examination is necessary for making the diagnosis of basidiobolomycosis. It shows a granulomatous reaction with giant cells and histiocytes in the central area of necrosis. The presence of thin-walled hyphae surrounded by eosinophilic infiltrate (Splendore-Hoeppli phenomenon) is a hallmark of basidiobolomycosis [5]. The treatment of cutaneous basidiobolomycosis mainly involves azole antifungals [3]. However, a case report showed the complete resolution of gastrointestinal basidiobolomycosis with the use of potassium iodide [10].

## Conclusions

Basidiobolomycosis is a rare but potentially fatal fungal infection that can mimic other diseases, making early diagnosis crucial. The non-specific clinical presentation, along with its ability to involve multiple organ systems, often leads to delayed or misdiagnosis, as seen in our case. Histopathological examination remains the gold standard for diagnosis, and awareness among clinicians is essential to ensure timely identification and appropriate antifungal treatment. Given the potential for rapid deterioration, as demonstrated in our patient, early recognition and prompt intervention are critical to improving outcomes and preventing fatal complications.
